# Machines, mathematics, and modules: the potential to provide real-time metrics for pain under anesthesia

**DOI:** 10.1117/1.NPh.11.1.010701

**Published:** 2024-02-22

**Authors:** Ke Peng, Keerthana Deepti Karunakaran, Stephen Green, David Borsook

**Affiliations:** a University of Manitoba, Department of Electrical and Computer Engineering, Price Faculty of Engineering, Winnipeg, Manitoba, Canada; b Massachusetts General Hospital, Harvard Medical School, Department of Psychiatry, Boston, Massachusetts, United States; c Massachusetts Institute of Technology, Department of Mechanical Engineering, Boston, Massachusetts, United States; d Massachusetts General Hospital, Harvard Medical School, Department of Radiology, Boston, Massachusetts, United States

**Keywords:** pain, analgesia, functional near-infrared spectroscopy, anesthesia, brain imaging, real-time, near-infrared spectroscopy

## Abstract

The brain-based assessments under anesthesia have provided the ability to evaluate pain/nociception during surgery and the potential to prevent long-term evolution of chronic pain. Prior studies have shown that the functional near-infrared spectroscopy (fNIRS)-measured changes in cortical regions such as the primary somatosensory and the polar frontal cortices show consistent response to evoked and ongoing pain in awake, sedated, and anesthetized patients. We take this basic approach and integrate it into a potential framework that could provide real-time measures of pain/nociception during the peri-surgical period. This application could have significant implications for providing analgesia during surgery, a practice that currently lacks quantitative evidence to guide patient tailored pain management. Through a simple readout of “pain” or “no pain,” the proposed system could diminish or eliminate levels of intraoperative, early post-operative, and potentially, the transition to chronic post-surgical pain. The system, when validated, could also be applied to measures of analgesic efficacy in the clinic.

## 1 Introduction

### 1.1 Case for “Pain” Under Anesthesia

The idea of “nociceptive” signaling under general anesthesia is not immediately intuitive. The anesthetic state is such that there is no movement (loss of reflexes) when the surgeon operates, and the patient is rendered amnesic for the event.^1^ Surgery-induced activations (e.g., from tissue damage or other noxious stimuli) of peripheral nociceptive pathways through C and Ad fibers produce changes in brain pathways (viz., sensory, affective, and modulatory) in both animal and human studies. The ongoing activation of pain pathways may result in peripheral and central sensitization resulting in hyperalgesia where response to stimuli is exaggerated. As noted by others, “not timing but duration and efficacy of an analgesic and anti-hyperalgesic intervention are most important for treating pain and hyperalgesia after surgery.”^2^ Furthermore, independent of peripheral activation of nociceptive pathways immunological activation relates to the brain state during surgical stress: “the anesthetized brain is still physiologically ‘awake’ and responsive to the sterile stressors of surgery.” ^3^

Thus, the question that arises is whether nociceptive signals during unconsciousness such as that occurring in general anesthesia, would be characterized as pain under the current International Association for the Study of Pain (IASP) criteria for the definition of pain: “An unpleasant sensory and emotional experience associated with, or resembling that associated with, actual or potential tissue damage, with the following addendums that relate to the topic at hand (a) Pain is always a personal experience that is influenced to varying degrees by biological, psychological, and social factors; (b) Pain and nociception are different phenomena. Pain cannot be inferred solely from activity in sensory neurons; (c) Through their life experiences, individuals learn the concept of pain; (d) A person’s report of an experience as pain should be respected; (e) Although pain usually serves an adaptive role, it may have adverse effects on function and social and psychological well-being; (f) Verbal description is only one of several behaviors to express pain; inability to communicate does not negate the possibility that a human or a nonhuman animal experiences pain”^4^ (IASP definition of pain).

Points a, b, and f of the IASP definition of pain, are notable in the context of general anesthesia. With respect to the surgical/anesthetic experience, other conditions may play a significant role in the responsivity of the pain system to tissue damage. For example, prior biological (disease) and psychological (e.g., catastrophizing) states may lead to increased intra-surgical, and post-surgical acute pain and chronic pain (pain present at >3 months following the surgical event). Thus, the basal socio-biological state confers a relative resistance (high or low) to the surgically induced tissue damage and inflammatory response at the time of surgery^5^^,^^6^ and post-surgery.^7^ However, since the patient is unconscious and cannot report pain as noted in (f) of the IASP definition, the inability to communicate does not negate the “pain experience” for which there is no clear definition in the pain literature. In support of this notion, current data suggests that nociceptive processes occur during surgery (evoked and ongoing pain), and sensory spinal (dorsolateral spinothalamic tract), thalamic (ventrolateral thalamic nuclei), and cortical (SI) pathways are clearly activated under most general anesthesia.^8^^–^^13^ So too are emotional circuits including frontal brain regions.^14^ The ongoing barrage of nociception is well known to induce central sensitization under anesthesia, which clearly should also consider alterations in the whole pain connectome and a response that affects clinical outcomes including more severe and persistent pain.^15^^–^^17^ Not to mention, autonomic responses such as increased heart rate, blood pressure and breathing are established clinical indicators of pain under anesthesia. As such, we argue that having a quantitative, easily acquired, objective method for measuring pain/nociception under anesthesia would allow for rational interventions to inhibit peripheral nociceptive drive and limit “anesthesia pain” or “unconscious pain” and likely diminish both acute and chronic post-operative pain. The rationale for this includes the notion that measures of pain/nociception in the awake state are the same as those in the anesthetized state, at least for inhalational agents, even with added opioid agents.^18^^,^^19^

In this review, we evaluate current effectiveness of analgesia under anesthesia and provide a primer on current methods for evaluating pain under anesthesia. Section 2 summarizes the nature of anesthesia and its role in analgesia, including an overview of general anesthesia and human brain connectivity, as well as a short overview of lack of anesthesia effects on brain connectivity. Section 3 reviews current methods for evaluating pain under anesthesia in the operating room (OR), summarizing a number of technologies, including functional near-infrared spectroscopy (fNIRS), in terms of their applicability and adaptability to determine pain under anesthesia. Section 4 reviews pain measures using fNIRS and Sec. 5 introduces a proposed algorithm to acquire and evaluate pain and pain-associated variables in real-time in the peri-surgical period – from pre-surgical, intra-surgical to postsurgical states. Figure 2 shows an overview of our approach to obtain real-time measures of pain in the OR. In Sec. 6, we consider various applications in the OR, along with problems and potential solutions, with attention to biomarker standards. As with any new technology there are practical and medical practice issues that must be taken into account including sensitivity and specificity, accuracy, acceptance in medical practice, etc.

## 2 Current Effectiveness of Analgesia Under Anesthesia

### 2.1 Surgery and Pain – A Brief Overview

An estimated 310 million surgical operations are performed worldwide annually.^3^^,^^20^ Surgery, including the routine procedures, “attacks” the nervous system through peripheral nerve damage and consequent inflammatory processes. Acute pain following surgery can occur immediately after surgery to up to 3-months post operation.^21^ Progression to moderate or severe chronic postsurgical pain (>3 months after surgery) occurs in 10%–50% of patients.^22^^–^^24^ A number of clinical predictors likely contribute to pain chronification, viz. intraoperative pain load, pre- and postoperative pain intensity, hyperalgesia/allodynia,^23^^,^^25^^–^^27^ as well as peri-surgical psychological factors, for example, pain catastrophizing^28^^–^^30^ discussed in more detail below. These reflect a combination of processes resulting in central sensitization. At present, a serious gap in postsurgical pain prevention is attributed to the lack of pain treatment provided during the perioperative period. During surgery general anesthesia provides a state of unconsciousness but not adequate ongoing analgesia, which is usually supplemented based on clinical evaluation; the latter is usually provided on a clinical basis without a validated objective marker that anesthesiologists can monitor.^8^^,^^31^

Most anesthetics used for surgery are inhalational volatile liquids, e.g., isoflurane,^32^ sevoflurane,^33^ etc. Apart from nitrous oxide ($N_{2}0$), inhalational anesthetics are not analgesic^34^ and indeed, may produce hyperalgesia under certain conditions.^35^ As such, analgesia is obtained through the use of intravenous analgesics (e.g., opioids). While concentrations and clinical utility of inhalational anesthesia can be relatively easily monitored, analgesia is more of a clinical judgment.

Data obtained from both human and animal studies point out that well-defined brain systems are involved in the brain’s response to pain/nociception.^36^ In animal models of acute pain (including mice, rats, and monkeys), regions activated while under anesthesia include sensory (e.g., S1, insula, thalamus), non-sensory (e.g., cingulate), and pain modulatory pathways (e.g., periaqueductal gray).^37^^,^^38^ In humans under general anesthesia, fMRI studies show that unconsciousness and reduction in working memory, cognition, and sensory processes are not from a lack of ascending signaling but due to halted inter-cortical integration.^39^

#### 2.1.1 General anesthesia and human brain connectivity – decreased conscious awareness, no complete blockade of pain

How general anesthetics affect brain systems is also how they may alter specific systems associated with the major components of anesthesia: hypnosis/loss of consciousness, amnesia, areflexia and analgesia? While these have been reviewed elsewhere, we try to differentiate and summarize the differences in these components and how they differ from potential ongoing pain under general anesthesia, even with no movement and an unconscious patient:

##### Hypnosis/loss of consciousness

Sedation is a cortical and thalamic function, disrupting higher cortical functions particularly the connectivity between the frontal and parietal cortices.^39^ Inhalational anesthetics seem to produce this disconnection, with little evidence of subcortical changes.

##### Areflexia

Pain usually causes a motor response. With general anesthesia, an additional drug is administered to produce blockade of the motor response to provide a non-moving patient for safe surgery. This of course diminishes the clinical evaluation of pain inducing procedures.

##### Amnesia

The induction of amnesia may modulate hippocampal function.^40^ As a clinical function it helps the anesthetic experience by making patients have no recall about the anesthesia.^41^

##### Analgesia

General inhalational anesthetics, for the most part, do not produce ongoing analgesia (with the possible exception of nitrous oxide.^32^ This is supported by functional imaging data in animals^13^^,^^37^^,^^42^ including non-human primates^43^ and humans^9^ as well as electrophysiological data.^44^^,^^45^ Some anesthetics (e.g., the $\alpha_{2}$-agonist, dexmedetomidine) may produce thalamocortical disconnection.^46^ However, non-inhalational anesthetics, including dexmedetomidine^47^ and ketamine,^48^ opioids,^49^ for example, do provide analgesia as part of their anesthetic effects. At sub-anesthetic doses, inhalational anesthetics may produce anti-analgesia or hyperalgesia effects.^35^

## 3 Current Methods for Evaluating Pain Under Anesthesia in the Operating Room

### 3.1 Clinical

Ever since the first use of ether as an anesthetic for surgery, clinical signs have been used to evaluate the analgesic status of patients undergoing surgery.^50^ Aside from analgesia, other signs include hypnotic state, muscle relaxation, and amnesia. With increasing complexity of anesthesia – from pharmacological agents to more sophisticated monitoring equipment – the “pain status” of the surgical subject remains a subjective evaluation by the anesthesiologist. Pain responses are directly related to the surgical depth of the patient. Clinical signs of the level of analgesia include respiratory movement, circulatory changes among others. In addition to the variability or lack of specific objective measures for pain *per se*, awareness during general anesthesia,^51^^,^^52^ where the patient is awake but paralyzed by anesthetic agents, contributes to the need for an objective measure for pain/nociception under anesthesia.

### 3.2 Physiological/Autonomic

Nociception is known to elicit the autonomic nervous system by modulating the sympathetic and parasympathetic systems, either reciprocally, independently, or in parallel.^53^ Thus, autonomic measures to nociception could provide potential surrogate markers of noxious activity during surgery. The measures may include parameters of the cardiovascular system [heart rate, heart rate variability (HRV), HRV derived analgesic nociception index, blood pressure, pulsatile component of cardiac cycle], respiration, skin conductance and pupil reflexes, etc. Cowen and colleagues provide a detailed review of these autonomic measures in awake and perioperative stages in relation to individual reported pain intensity levels.^31^ Physiological measures are non-invasive, easy to acquire and interpret, and are routinely implemented during surgery (electrocardiography and blood pressure). However, the primary limitation of these techniques is the lack of sensitivity and specificity to pain, as well as the potential influence of anesthesia and analgesia on the autonomic state. More recent studies show that pain appraisal, expectation, anticipation, and affect can also influence autonomic response to noxious activity.^54^

### 3.3 Electroencephalography (EEG)

In the past decades, the use of an EEG-based device, such as the bispectral index (BIS) monitoring system, to monitor the anesthetic condition of surgical patients has been more widely accepted in the OR.^55^ This method generally employs a few EEG electrodes to measure electrical signals from the frontal part of the brain. The EEG-derived indices or parameters are based on state-of-the-art temporal, frequency, and phase analyses of recorded electrical signals, which correspond to the levels of patient consciousness at different concentrations of hypnotic drugs including both propofol and volatile agents (such as isoflurane or sevoflurane). These indices are used to guide intraoperative anesthetic titration – from patient full wakefulness to light anesthesia but to a large extent do not convey enough information on the noxious stimulations during surgical procedures. For example, when noxious stimuli are applied, the BIS index can increase in some patients, decrease in some other patients, or stay unchanged.^56^ Instead of relying on post-processed indices, several studies have recommended looking at raw electroencephalograms to identify changes in EEG band powers following noxious stimulations.^57^ Patterns that are more frequently seen include an increase in EEG beta power (“beta arousal”), an increase in EEG delta power (“delta arousal”), and a reduction in EEG alpha power (“alpha dropout”). Many researchers argue that maintaining maximum alpha activity during surgical nociceptive inputs would be the key for adequate analgesia.^58^^,^^59^ However, most of them would also agree that a major limitation of such idea on “analgesic titration” is the significant variations of raw EEG and alpha power across individuals, which largely depend on factors such as age and patient cognitive conditions.^60^ The administration of analgesic drugs to revert the EEG changes and to restore the previous state is therefore inherently patient- specific. Table 1 compares the advantages and limitations of the various techniques including other neuroimaging techniques such as fMRI.

**Table 1:** Advantages and disadvantages of each modality for objective pain measures in the OR.

Domain	Advantages	Disadvantages
Physiological	Portable	Practicality and complex: pupil oximeter, CARDEAN index, photoplethysmography
	Non-invasive	
	Easy to use	
	Many are already routinely used in the OR	Non-specificity: heart rate variability, blood pressure, skin conductance
	Compact	
		Influenced by anesthetics/analgesics: plethysmography, heart rate variability, blood pressure, etc.
EEG	Portable	Signals are complex and need pre-processing
	Non-invasive	
	Directly measures signals from the central nervous system	Most systems measure “level of consciousness” rather than nociception
	Several monitoring systems based on EEG are already available in the OR (such as the BIS system)	Current findings on EEG changes associated with nociception have significant variations across different subjects.
fMRI	Details of whole brain function including connectivity	Non-portable
	Non-invasive	More difficult to achieve real-time data
	Measures of multiple brain functions	
fNIRS	Portable	Superficial measures of cortical brain function
	Non-invasive	
	Faster acquisition of data versus fMRI	Moderate spatial resolution (2 to 3 cm)
	Correlation of functional and anatomical data	
	Real-time data achievable for decision processes in the OR	

## 4 Functional Near-Infrared Spectroscopy (fNIRS) – A Potential New Era

One of the challenges of defining an “objective measure” is the specificity of the method. Since this may fall under the rubric of “biomarker,” which as defined by the National Institutes of Health (NIH) Biomarkers Definitions Working Group is “a characteristic that is objectively measured and evaluated as an indicator of normal biological processes, pathogenic processes, or pharmacologic responses to a therapeutic intervention.”^61^ Based on this definition, we provide an approach to measure pain under anesthesia and have a real-time measure of evoked and ongoing pain that can be quantitatively presented and used to “sound the alarm” for a therapeutic intervention. Below, we present evidence in support of an objective brain-based pain assessment tool using functional near-infrared spectroscopy (fNIRS).

### 4.1 Technology

fNIRS is a noninvasive neuroimaging technique that utilizes near-infrared light (∼690 to 850 nm) to provide a continuous measure of cortical hemodynamics viz. oxygenated hemoglobin (HbO) and deoxygenated hemoglobin (HbR) concentration changes.^62^^–^^64^ With a similar physiological basis as functional magnetic resonance imaging (fMRI), fNIRS shows significant advantages for being robust, portable, and cost-effective. However, fNIRS is also limited by its sampling depth (i.e., <1  cm within the cortex) and its susceptibility to hair contaminations.^65^^,^^66^

### 4.2 fNIRS Measures of Pain Neurotransmission

**Fig. 1:**
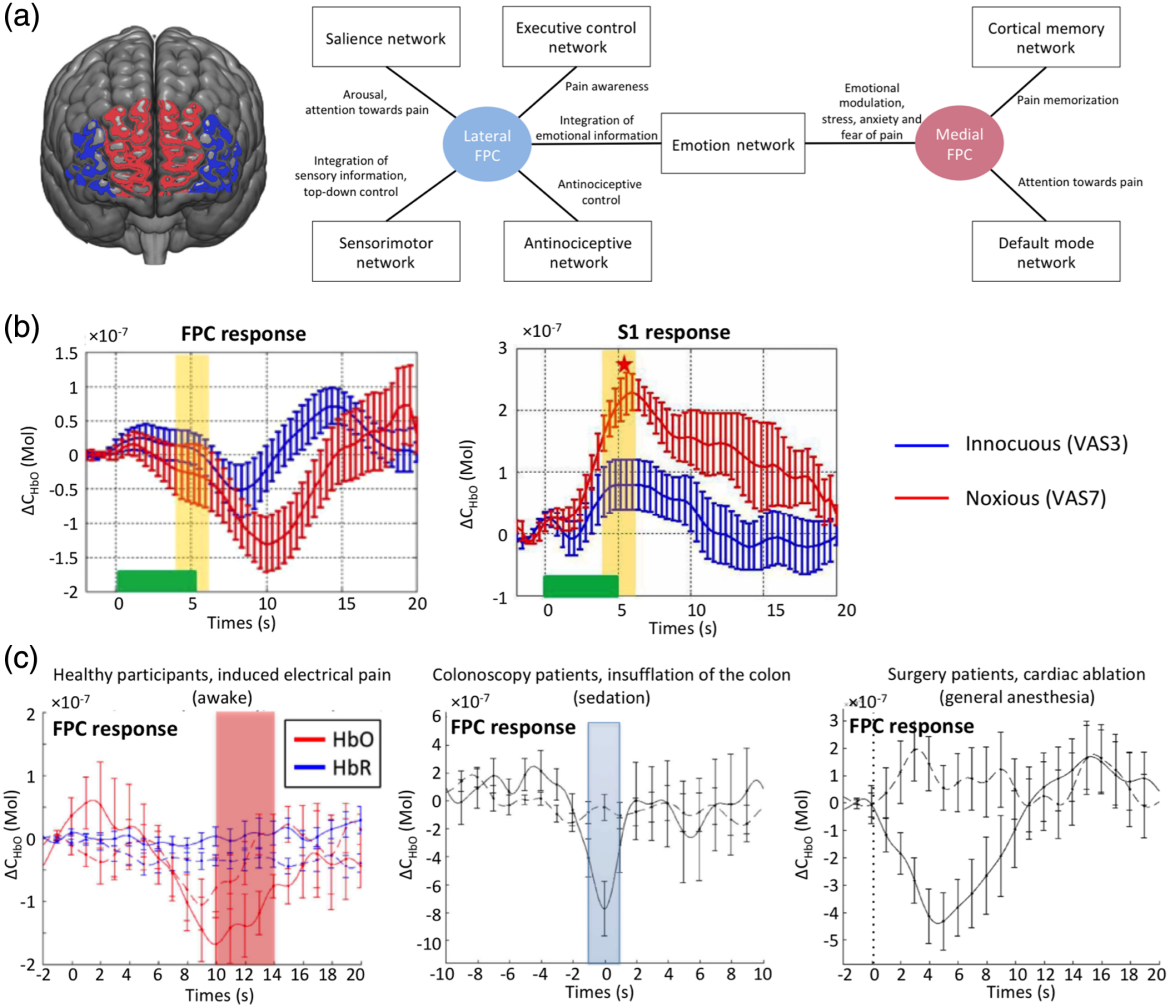
fNIRS signals from brain regions and responsivity to acute painful stimuli: (a) Medial versus lateral polar frontal cortex. Left: Depiction of the anatomical locations of the medial FPC and the lateral FPC. Right: Overview of the connections and functional roles of the FPC in the processing of pain, adapted from Peng et al.^14^ (b) Hemodynamic response of the medial frontopolar cortex and S1 during five seconds of innocuous (VAS 3/10) versus noxious (VAS 7/10) electrical stimulation in 11 healthy volunteers. The error bars indicate the standard error of mean. Figure adapted from Yucel et al.^81^ (c) Comparison of Oxy-hemoglobin and deoxy-hemoglobin concentration changes to electrical pain (left), insufflation (middle) and cardiac ablation (right) under awake, sedated, and anesthetic states, respectively. The error bars indicate the standard error of mean. Montage adapted from Becerra et al.,^82^ Kussman et al.,^83^ and Aasted et al.^84^

fNIRS, like fMRI, is based on the principle of neurovascular coupling to link neuronal activity and cerebral blood flow and blood volume changes. fNIRS-derived pain measures from cortical regions reflect the system level processing of nociceptive activity on the premise that (1) the primary sensory cortex is a major integrator of sensory afferent information including pain/nociception; (2) its connectivity to other brain regions, in our case, the polarfrontal regions, may be specific to pain state. Our past work to establish a measure for pain/nociception under general anesthesia, focused only on the cortical areas of the frontopolar cortex (FPC, which is the anterior portion of the prefrontal cortex) and primary somatosensory cortex (S1). We focused on the FPC and the somatosensory regions because of the specificity of the somatosensory cortices in nociception and the potential of measuring a signal indicating the integration of nociceptive and higher-order information at the prefrontal cortex that has not been blocked with anesthetics.^67^^,^^68^ Under deep anesthesia, connections between networks are disrupted and although nociceptive information reaches the sensorimotor network, its propagation and integration in prefrontal areas does not take place.^39^ The primary somatosensory cortices are classic sensory processing regions that are well known to be involved in the encoding of sensor – discriminative information of pain.^69^^,^^70^ The S1 receives nociceptive/pain information from second-order neurons along well-defined nociceptive pathways viz., spino-thalamo-cortical tract.^71^ Numerous studies including electrophysiological,^72^^–^^74^ anatomical,^70^ brain imaging,^67^^,^^68^ and clinical observation^75^ support this. Furthermore, SI activation is seen in response to C and A-delta fibers^76^^,^^77^ as well as to A-beta fibers by mechanical stimulations.^78^ In addition to activation of this area by painful stimuli, diminished activation is observed in S1 following analgesic treatment (e.g., fentanyl)^79^ or lesion (e.g., of the spinothalamic tract).^80^ fNIRS activation by non-noxious stimuli (i.e., brush produces an activation in the SI region that can be differentiated based on their amplitude and profile.^81^ The putative roles of frontal regions in pain have been reviewed by us elsewhere.^14^ Based on the anatomical and functional connectivity and the putative roles, we have proposed that the FPC could at least be parceled into two distinct subregions, i.e., the lateral portion of the FPC (lateral FPC) and the medial portion (medial FPC), see Fig. 1(a). The processing of pain information in the brain may involve both subareas, however each may be associated with distinct brain functions. Their functional differences may be reflected by their distinct afferent/efferent pathways and anatomical connections, as well as through the co-activation or co-deactivation of remote brain areas as large-scale brain networks.^14^ In particular, the lateral FPC is a part of the “salience network” and may play an important role in the switch of the brain from an “internally focused” state to an “oriented attention” toward pain.^85^ Network connections may potentially provide a functional pathway for the lateral FPC to interact with the sensorimotor network for the integration of the sensory-emotional information.^86^^–^^88^ Through efferent projections to regions such as the periaqueductal gray,^89^ the lateral FPC may execute top-down control (inhibit under healthy conditions) to modulate nociceptive activation of cells in the dorsal horn of the spinal cord.^90^ The medial FPC, as a key hub in the default mode network, may be under the control of the salience network during the attention switch. Moreover, the involvement of the medial FPC together with the anterior cingulate cortex in the context of affective-motivational processing of pain (such as stress, anxiety, and fear) has also been reported in many studies. In fNIRS studies of pain/nociception, FPC response is time-locked with the primary somatosensory cortex activation and is anticorrelated.^19^ We have previously reviewed connections between SI and BA10 including the polar frontal cortex.^14^ Data across different activation measures indicate that fNIRS measures are similar to fMRI studies.^91^^–^^93^ Figure 1(c) shows an overview of the potential distinct functions of both the lateral FPC and the medial FPC in pain processing. We propose to measure brain signals from both FPC subregions using fNIRS.

### 4.3 Measure of Acute Pain Using fNIRS

The hemodynamic response to acute pain has been well characterized for the somatosensory and frontal lobes through our ongoing work. Findings from our prior fNIRS datasets are consistent with a nociceptive pain signature being measured.^81^^–^^84^^,^^94^^–^^96^ Acute pain measures are important in the anesthesia setting as they relate to measures of specific nociceptive inducing procedures (e.g., cutting, cauterizing, scraping, stretching, etc.). In Fig. 1(b), we show the inverse (anticorrelated) relationship between fNIRS-measured acute pain signals in the primary somatosensory cortex (activation) and the FPC regions (deactivation). This functional dissociation is characteristic of nociceptive signaling to the brain. Moreover, this functional response to acute pain produced a similar response in the frontal region across different levels of consciousness [Fig. 1(c)].

Principle of two brain regions involved in sensory and emotional/cognitive nociceptive/pain processing are anticorrelated:^81^ This concept has been further defined by understanding differences in fNIRS signals in the medial and lateral polar frontal cortex (see Ref. 14). We have focused on the polar frontal and somatosensory regions because of the specificity of the somatosensory cortices in nociception and the potential of measuring a signal from the polar frontal areas indicating integration of nociceptive information in frontal regions that is not blocked with anesthetics. Previous evidence has suggested that while there is a breakdown of brain functional connectivity during anesthesia for most systems, this is not the case for sensory connections.

Frontal Lobe Signal During Painful Stimuli: Evoked noxious stimuli produced a consistent pattern of HbO changes (solid line) in the frontal lobe response at three different states of consciousness (awake,^84^ sedated,^82^ and anesthetized^83^). A common signal is observed in the medial frontal lobe that is present with a predetermined noxious signal. For the anesthetized state, subjects received cardiac conduction ablations and show consistent results. The frontopolar cortex is potentially at the top of brain hierarchical structures involve in the pain connectome. In a recent review of frontopolar changes in pain across multiple brain imaging approaches we summarize the region’s role in pain processing.^14^ Activation in the medial FPC is observed in both acute and chronic pain.^84^^,^^95^^,^^97^^–^^99^ The medial FPC is intimately connected to the brains default mode network, which we believe is a potential target to evaluate a measure of ongoing pain in patients based on changes in high and low frequency oscillations derived from the fNIRS signal.^11^ As recently reviewed by our group, evidence suggests that BA 10, and particularly the medial FPC, may play a critical role in the collation, integration, and high-level processing of nociception and pain but also reveals possible functional distinctions between the sub-regions of BA 10 in this process.^14^

### 4.4 Measure of Ongoing Pain Using fNIRS

Our published data have shown the potential of measuring continuous or ongoing pain/nociception using fNIRS-measured FPC signals in awake volunteers as well as anesthetized patients.^11^^,^^100^

**Fig. 2:**
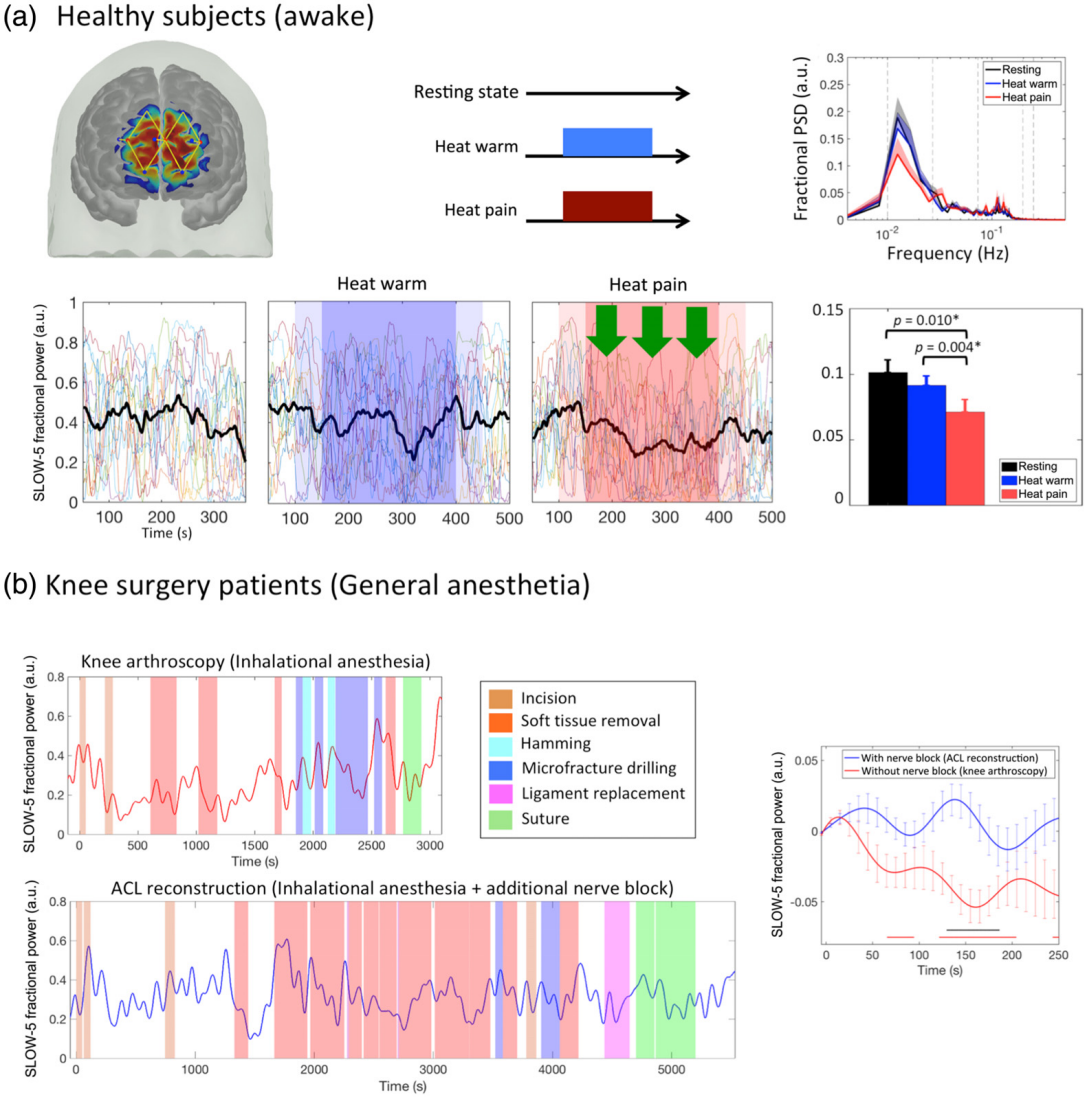
Cortical power as a measure of ongoing nociception/pain:^11^ (a) Power spectral analysis of the prefrontal cortex signal at SLOW-5 frequency range (0.01 to 0.027 Hz) revealed a drop in power during periods of noxious stimulation (heat pain) that was not found during innocuous stimulation (heat warm) or resting state. Bar plot shows a decrease in mean fractional power during pain when compared to no-pain (p=0.004) and resting conditions (p=0.010). (b) Similar decrease in cortical power was observed during periods of potentially painful surgical procedures in a patient undergoing knee arthroscopy under anesthesia. This was performed using dynamic power spectral analysis, which is an extension of the method described in (a). Potentially painful procedures are indicated by the color shaded regions. The error bars indicate the standard error of mean.

By converting the FPC signals into the frequency domain using an fast Fourier transform (FFT), we have identified a reduction in power in the SLOW-5 frequency band (0.01–0.027 Hz) in response to ongoing painful stimuli (Fig. 2). Ongoing pain becomes a critical measure in evaluation of perioperative pain as the first set of surgical procedures likely initiate both peripheral or central sensitization^101^ leading to a sensitized state and spontaneous intermittent or continuous nociceptive signaling that persists into the post operative period that acts on the sensitized brain/spinal cord that may be a likely harbinger of chronic pain following surgery.^16^^,^^102^^,^^103^ The practical application of the frontopolar cortex slow-5 signal to effectively capture ongoing nociception may necessitate further research for comprehensive validation across a broader spectrum of stimulus types or clinical conditions.^11^ This entails evaluating its sensitivity and specificity profile, along with the development of analytical methods capable of facilitating real-time processing of dynamic signal features. Future work is essential to validate and refine the utility of the frontopolar cortex slow-5 signal, ensuring its reliability and applicability for more robust nociception monitoring in this context.

### 4.5 How Do We Know the Signals as Measured by fNIRS Reflect Nociception/Pain

**Fig. 3:**
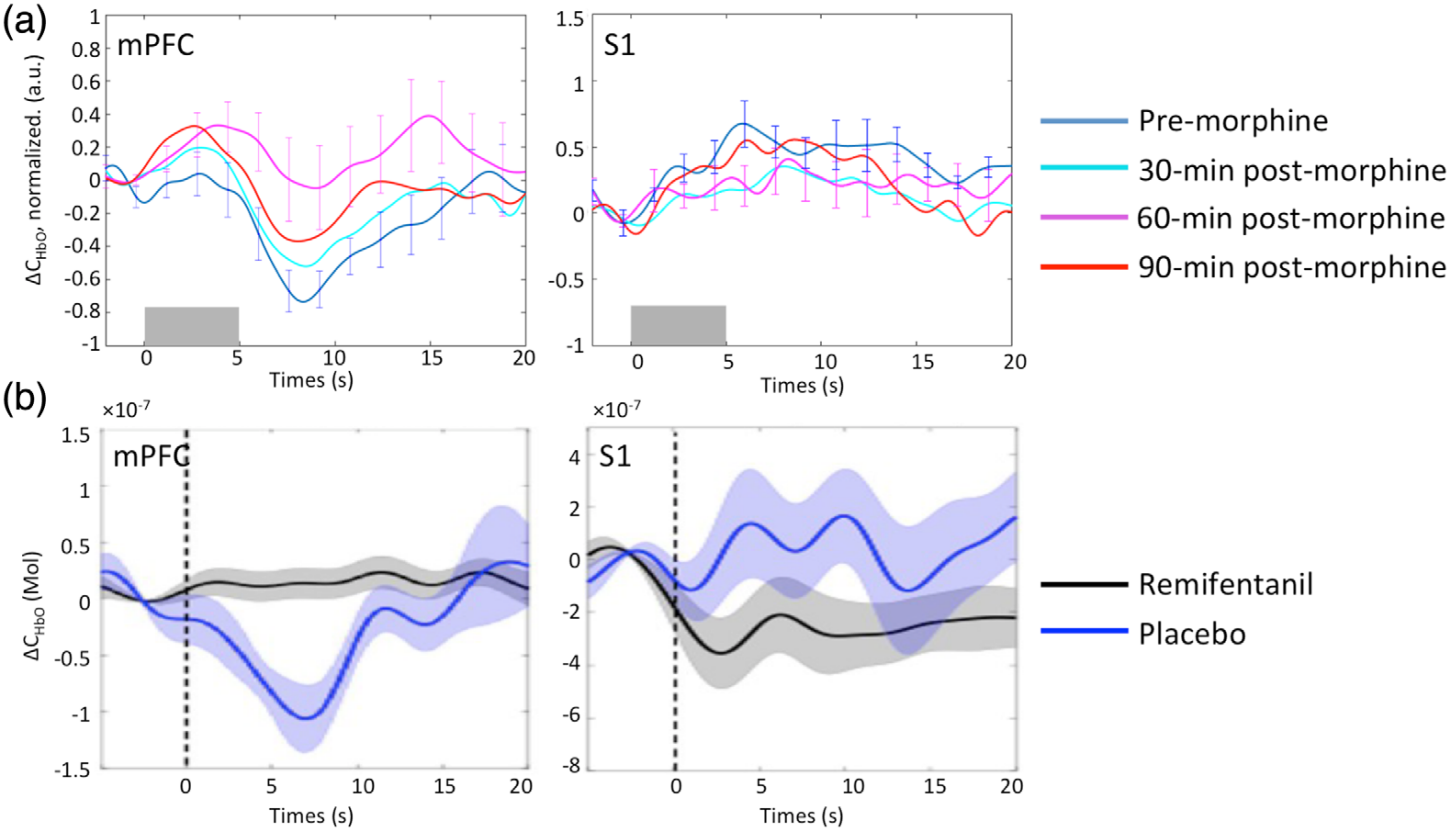
Modulation of mPFC and S1 responses to evoked pain stimulations by analgesic measures: (a) Oral morphine reduced the amplitude of mPFC HbO decrease and S1 HbO increase associated with electrical pain stimulations in healthy, awake volunteers.^96^ The extent of amplitude reduction was consistent with the pharmacokinetic-pharmacodynamic model of oral morphine. Curve legend – blue: at baseline (pre-morphine); cyan: at 30-min post morphine administration; magenta: at 60-min post morphine; red: at 90-min post morphine. The gray horizontal bar indicates the timing of electrical pain stimulations. (b) Compared with placebo (blue curve). The administration of remifentanil (black curve) reduced the mPFC and S1 responses to invasive surgical procedures (cardiac ablation) in patients under general anesthesia.^94^ fNIRS-derived cortical response is attenuated by opioids. Hemodynamic response of mPFC and S1 regions was attenuated by morphine but not placebo in awake healthy volunteers (a). Similarly, both mPFC and S1 response was attenuated by remifentanil but not placebo in anesthetized patients undergoing cardiac catheter ablation. The error bars indicate the standard error of mean.

Our measure (inverse correlation between mPFC and SI) is consistent with a marker that is the (a) same across different states of consciousness, (b) is attenuated by opioids (Fig. 3), and (c) can be differentiated from non-painful signals (e.g., auditory) including graded responses for low versus high pain intensities [Fig. 1(b)].^18^^,^^100^^,^^104^ While there are issues of overlap in fNIRS brain responses to categories of stimuli or states (e.g., aversive), the reproducible response during awake and anesthetized states suggests these are not influenced by emotional valence, anticipation, unpleasantness, etc. Furthermore, our current and prior data define the characteristics of an fNIRS mPFC signal that (a) is specific in the context of stimulus type such as experimental or clinical persistent pain and differs in responding to these pain stimuli and to non-painful sensory (brush) or rewarding/aversive pictures (psychological stimuli);^81^^,^^84^ (b) can evaluate both acute and persistent pain in the OR;^11^^,^^83^ (c) is reversed by analgesics;^96^ (d) is consistent with the literature (predominantly fMRI) of pain imaging using frequency analysis; specifically, low-frequency fNIRS oscillations are similar to those acquired using fMRI to reflect pain states by us^105^ and others;^106^^–^^109^ and (e) shows expected changes in central sensitization. Finally, other groups have utilized fNIRS to measure pain/nociception (see Refs. 19 and 110 for recent reviews).

## 5 Pain Measures Using fNIRS – An Algorithm

**Fig. 4:**
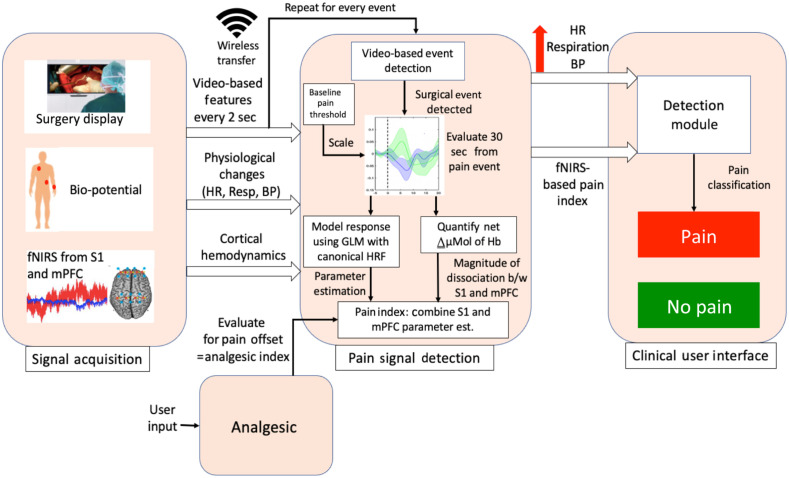
Summary of set-up for the anesthesia pain monitoring procedure (APMP): The figure shows an overall approach to real time measures of pain (i.e., on or off) during surgery based on video-based detection algorithm. The surgical display can be connected to the system to identify potentially painful events or surgical manipulations to feed the algorithm, which will then evaluate the fNIRS and physiological data to calculate spatial and temporal features relating to that event. These features will be inputted into the classifier to perform a binary classification of Pain versus No PE.

In the sections that follow below, we integrate our approach to an algorithm for an objective measure of pain in the perioperative period. As shown in Fig. 4, the process involves multiple processes related to measures of fNIRS data acquisition, physiological monitoring, real-time data analysis, and mathematical models of determining the analgesic status (pain alert and pain off) of the patients. Specifically, as detailed below, the approach allows a reasonable time delay between recorded hemodynamic signals and outputted pain measures and therefore necessitates the implementation of a real-time framework for data processing. We propose to apply a sliding window method, where a data buffer is created and used to store the recorded optical data of all available channels over a defined time period. As new data are collected, the data buffer is updated at each time point by discarding the earliest data points and connecting the new data points to the end of the buffered time courses. Updating the buffer triggers a series of processing steps that are described below. Additional inputs to improve the sensitivity of the system include a user-defined input of analgesics administered prior to or during the procedure, baseline pain threshold of the patient as determined prior to surgery, and use of surgery display as input to the algorithm to identify surgical events and the related biological (brain and physiological response).

### 5.1 fNIRS Setup

**Fig. 5:**
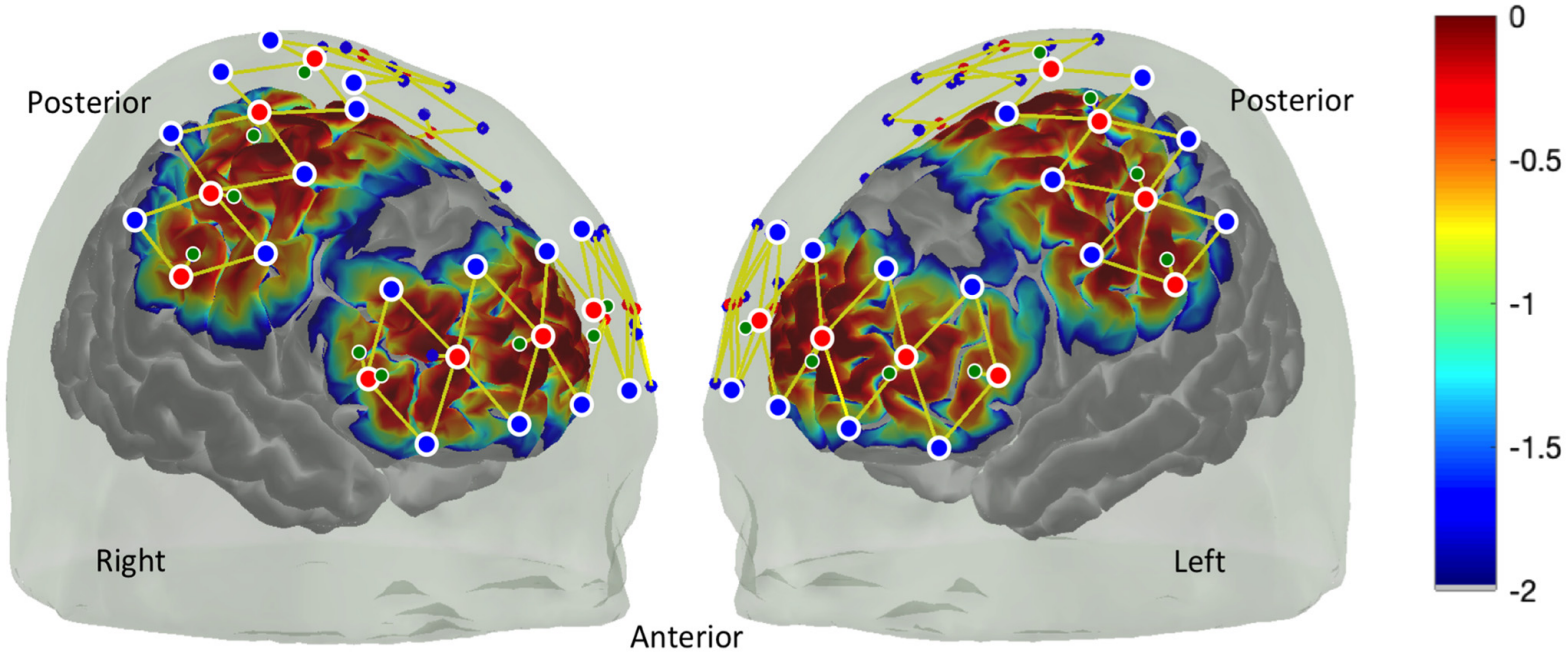
Placement of fNIRS emitters (red) and detectors (blue) for surgery and corresponding cortical sensitivity: Green dots show short separation detectors for the purpose of removing global physiological effects. Warmer colors indicate high sensitivity and cooler colors indicate areas of low sensitivity.

Simultaneous recordings of hemodynamic signals from two cortical regions, i.e., the S1 and the FPC, are used. These regions can be assessed by fNIRS with a reasonable sensitivity and reliability.^111^^,^^112^ S1 is a classic region known to be within the lateral (sensory) nociceptive pathway while the FPC is known to be involved in the high-level integration and processing of nociceptive information.^14^ To better characterize the physiological components in the fNIRS signals, we propose to apply short separation detectors, which are light detectors placed at a distance of <1  cm from the light emitters to capture hemodynamic signals from extracerebral layers (e.g., skin, scalp, and skull). Figure 5 shows an example setup of the fNIRS optodes on the patient head and the corresponding cortical sensitivity profile.

### 5.2 Modules and fNIRS for Anesthesia Pain Monitoring Procedure (APMP)

**Fig. 6:**
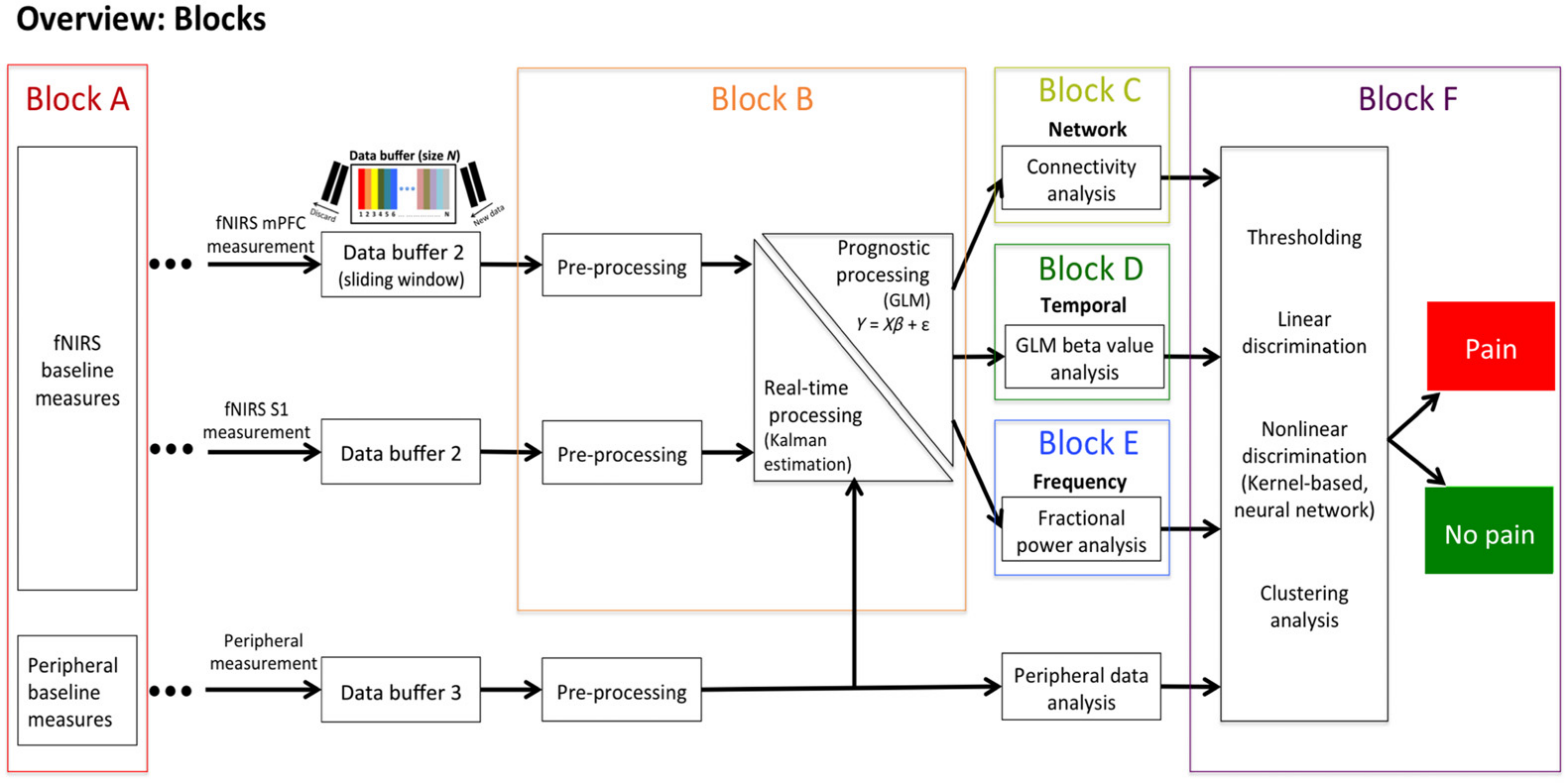
Overview of data processing pipeline. Block A – Baseline measures; Block B – fNIRS data pre-processing; Block C: functional connectivity analysis; Block D: GLM beta value analysis; Block E: time-frequency analysis of low frequency oscillation fractional power; Block F: Integrative classifier for pain/no pain classification.

In our formulation of automated data collection and analysis for pain measures under anesthesia, a number of steps needs to be integrated and these are shown in Fig. 6. Each of these modules in Fig. 6 are defined as “blocks” and discussed in detail below.

#### 5.2.1 Block A – baseline measures

fNIRS optodes and physiological sensors are installed on the patient’s head and body (Fig. 5) in the pre-surgery room. After the setup, anesthesiologists and nurses conduct medical interventions such as anesthesia procedures or drug administrations to prepare the patient for the surgery. After further adjustments and validation of fNIRS measures to ensure good optode contact and signal-to-noise level, we propose to perform a baseline scan of 2 to 10 min to establish a dataset of resting state signal parameters regarding functional connectivity strength, beta values from general linear model analysis and low-frequency fractional power from signal frequency analysis (see the following blocks for respective analyses) (Fig. S1 in the Supplementary Material). These form the patient-specific baseline to compare acute or ongoing events. The fNIRS data acquisition continues as the patient is transferred from the pre-surgery room to the operation room to receive the surgery.

#### 5.2.2 Block B – fNIRS signal pre-processing

The fNIRS data are recorded continuously until the end of the surgery. At each time point (defined by the sampling time of the fNIRS system – e.g. every 0.04 s), the data buffer is updated, and the buffered data are first pre-processed (Fig. S2 in the Supplementary Material). The raw optical intensities are first converted into optical density changes by taking the logarithm of the time course. Motion artifact detection and correction are performed on the converted optical density data using both signal amplitude and variance thresholds. For artifacts that are excessive and cannot be corrected within a few iterations, the entire data in the buffer are discarded. The motion-corrected optical density time course then undergoes bandpass temporal filtering (e.g., with typical low-pass threshold = 0.5 Hz and high-pass filter = 0.01 Hz) to remove signal components that unlikely have a neurophysiological basis. The filtered optical density data are then transformed into hemoglobin concentration changes using the modified Beer–Lambert Law. To better characterize the physiological components in the fNIRS signals, we use short separation detectors, which are light detectors placed at <1  cm from the light emitters. The short separation regression method has been shown to be effective in removing the hemodynamic signals from extracerebral layers (e.g., skin, scalp, and skull) from fNIRS-measured hemodynamic signals.^113^^,^^114^ For each channel, a linear model can be set up to regress extracerebral contaminations and other physiological noises using the time course of the short distance detector that has the highest correlation with the channel time course as the regressors. The pipeline may be customized and modified to suit the study paradigm, including the modification of filtering frequency range, use of the nearest short separation channels for temporal regression, principal component analysis-based noise reduction, etc.

#### 5.2.3 Block C – connectivity analysis

One of the measures/features of the classifier is based on the nociceptive signature we have observed in several of our past fNIRS datasets. The functional dissociation between mPFC and S1 hemodynamic response to acute pain was unique to noxious stimuli and diminished following opioid administration. Such functional relationship between brain regions may be calculated using functional connectivity, defined as the statistical dependencies between signals across time. These connectivity measures, when evaluated across spatially distant anatomical regions, will allow the study of the brain as a network. Network connections, also known as connectome, can be unique markers of individual,^115^ trait, state, disease, etc. In this case, the functional connectome between the prefrontal cortex and somatosensory and perhaps other regions may be used as a nociception signature under general anesthesia. However, since surgery is a dynamic event where surgical events may be considered analogous to exogenous task stimuli, we propose a sliding-window correlation technique, where we calculate the pairwise correlation of the two regions over smaller time windows^116^^,^^117^ (Fig. S3 in the Supplementary Material). The preprocessed time series from the regions of interest (mPFC and S1) will be correlated using Pearson’s r correlation within an overlapping window of 20 s duration to generate a log of dynamic functional connectivity. Additional window lengths of 10, 30, 40, 50, 60 s durations can be used to generate secondary dynamic functional connectivity logs in parallel. The Pearson’s r correlation values are then converted to Fisher-z scores using Fisher r to z transformation before inputting into the classifier.

#### 5.2.4 Block D – general linear model beta value analysis

The functional dissociation between mPFC-S1 may also be evaluated by comparing the activation levels of mPFC-S1 as opposed to FC (Block C). General linear model using a canonical HRF can quantify the activation level in response to acute events, although a negative HRF (denoting deactivation) will be necessary to model the mPFC response. By extending the dynamic FC approach, we propose a GLM based dynamic evaluation of activation levels across time i.e., surgery (Fig. S4 in the Supplementary Material). The activation level (beta estimates) and other parameters of GLM at single-window level will be compared between the mPFC and S1 regions. Based on our hypothesis, a functional dissociation resulting from acute surgical pain will be reflected by (a) negative activation in mPFC and positive activation in S1 as modeled using pre-specified HRFs, and (b) low levels of Sum of squared errors in both mPFC and S1 regions. The use subject-specific HRF’s using a prior run or the use of the first surgical event as a subject baseline can improve the accuracy of this technique further.^118^ These estimates will be recorded over time (to generate a GLM log) and inputted into the classifier along with physiological responses to detect periods of evoked painful activity.

#### 5.2.5 Block E – frequency analysis

The buffered hemoglobin concentration time courses are first converted into the frequency domain using the FFT (Fig. S5 in the Supplementary Material). Power spectral density (PSD) is computed as the square of the FFT amplitude at each frequency component. The fractional power spectral density (fPSD) of the slow-5 sub-band (i.e., 0.01 to 0.027 Hz) is extracted by adding the PSD components of the slow-5 sub-band and normalized to the summed power of the entire frequency range (0.01 to 0.5 Hz). The fPSD of slow-5 is then transferred to the classifier as an input for pain/nociception classification.

#### 5.2.6 Block F – pain classifier

Once each of the N chosen parameters have been collected, distinctions can then be made within real time between the current signal that is analogous to the first pain event (PE) as opposed to a non-painful baseline period, flagging events of concern to the end user. This requires a binary classification, where using a fixed number of features (which may vary from n=1 to N), the algorithm classifies a given surgical period as pain or no pain state. Considering the real-time nature and limited number of samples for training, we require a classifier with high accuracy, that is computationally efficient with low memory requirements, and can continuously update with real-time data. A feature selection may be performed using a filtering or embedded algorithm, however, we propose to assign a multiplier value or weighting factor that determines how relevant to the categorization approach the end user wants it to be for the algorithm (Fig. S6 in the Supplementary Material). Based on prior data from our group and others, cortical-based features will be assigned greater weightage than physiological data. Alternatively, this can be set to 0 and ignored entirely if so desired. The result can undergo a form of dimension reduction such as PCA or linear discriminant analysis (LDA) to create a component matrix X in a reduced set of dimensions. Although, there is a risk of overfitting the data when using a lot of features. From here, we could implement a linear classifier such as logistic regression. Logistic regression uses a linear combination of the predictive features to generate the probability of a PE. At least one to three PEs conducted initially by the surgeons are recorded for a duration equal to the chosen time window (TW). A series of TW’s are also observed for the baseline region where no pain events (NPE) have been conducted. These are the only training datasets available for a classifier. Thus, classifier methods such as support vector machine may be computational taxing and unsuited for real-time monitoring in the OR.

Alternatively, we could implement a rule-based learning classifier. Pearson correlations can be taken between the variables that make up the component matrix X for the current time t and the series of pain and no pain TW’s. If most of these correlations are shown to be more positive toward the series of PEs than the baseline events, an adverse event can be recorded. This can be defined in the equation $\mathrm{IF}\;\frac{\sum_{x,y=1}^{N}\;\mathrm{IF}\;\mathrm{corr}(X({\mathrm{CT}}_{x,y}),X({\mathrm{PE}}_{x,y}))>\mathrm{corr}(X({\mathrm{CT}}_{x,y}),X({\mathrm{NPE}}_{x,y}))}{N}>\mathrm{CI}\to P=P+1$, where the IF statement in the numerator provides a boolean answer and the confidence interval CI is a value between 0 and 1 (0.9 and 0.95 are common examples of potential intervals). If this condition is met, the number of recorded PEs P is updated with the current time point t recognized as a PE.

Currently, the fNIRS process we propose could provide a response signaling that pain is occurring during the evoked stimuli and ongoing background index of pain. Specific quantification of the level of pain has not been evaluated today since amplitudes may vary across individuals due to anesthetic levels, premorbid psychological status, premorbid pain status, etc. The approach we have used applies a threshold above which “pain/nociception” is determined with confidence. More sophisticated approaches are likely to be developed in future, for example, measures of amplitude, frequency, or time-frequency or spectral measures that indicate levels of pain intensity or resolution.

## 6 Clinical Use – Adoption Process using Biomarker Standards

### 6.1 Disrupting the Status Quo – Becoming a Standard in the OR

There is a clear need for an objective marker/biomarker for pain.^119^^–^^121^ Such efforts have been supported by NIH with the view that objective methods allow for better analysis of disease state and/or treatment efficacy. The proposed system will also set precedence for any future autonomous biomarker-based pain mapping and further studies, with training providing finer ranges for when pain occurs and how surgeons are informed in the OR by the monitoring procedure.

The operating room is a complex and dynamic environment that is significantly limited on time. Therefore, any adaptations to the routine clinical care would have to be quick and easy to setup for clinical staff to operate. The proposed framework involving NIRS-based brain monitoring and its components can be easily integrated into the surgical setup due its small size and portability. Some of the physiological data such as heart rate, heart rate variability, respiration, etc. is also collected as part of clinical monitoring. These physiological sensors could be used simultaneously with fNIRS, also known as systemic physiology augmented fNIRS (SPA-fNIRS).^122^^,^^123^ SPA-fNIRS records physiological signals in tandem through equipment such as bracelets, patient monitors, and modules to measure systemic physiological activity such as heart rate, heart rate variability, mean arterial pressure, partial pressure of oxygen, etc. Some commercially available NIRS systems (NIRSport2, NIRx Inc.) are equipped with biosensors that can be time-synchronized with the fNIRS device. However, integrating the fNIRS-based system with the clinical monitors will simplify the setup in the operating room. As a light-based technique, the fNIRS system is unlikely to interfere with other electronic equipment or impose a radiation risk to the patient. Moreover, fNIRS devices have a long track record of being used to monitor cerebral oxygen saturations during cardiac surgeries within a complex operating room environment^124^^–^^126^ as well as in several non-cardiac surgery models.^127^ Our framework, leveraging the same imaging technique, could seamlessly be incorporated into existing practices (e.g., as an “upgrade” of the current systems), without imposing cumbersome procedural changes.

### 6.2 Specificity and Sensitivity

#### 6.2.1 What concerns are there regarding the specificity and sensitivity of the approach?

This is indeed a major issue in the field of pain. While we agree that the medial FPC fNIRS signal is not specific for pain, the signal responding to the specific evoked or ongoing stimulus (i.e., pain) is consistent with the sensory (nociceptive) and emotional (aversive) nature of pain and is context specific. Thus, although it is currently not possible to state that this is a specific pain signal, we feel confident that the proposed signal can be used as a good surrogate marker of nociception/pain as previously used for other applications such as disease diagnosis. Pain signals produced by the brain can take on many forms based on intra-patient variability. The proposed system will have to be fine turned to capture as many of these pain markers as possible, being sensitive enough to catch outlier events but specific to the point of not flagging any false positives that could occur in the OR.

#### 6.2.2 Subject variability: age, skin color and other physiological variables need to be considered

Dark skin color may alter the quality of fNIRS signal^128^ – notably the SNR is reduced. Our short source separations approach takes into account extracerebral systemic confounds to brain hemodynamic data. Skin melanin content can alter the fNIRS signal and this needs to be taken into account (viz., a compare data from dark skin versus light skin). We will use a threshold for the light intensity and intensity below a certain level will indicate optical uncoupling. Sensors will also be affected by any hair on the subject’s scalp and will need to be considered before future procedures are conducted. Further factors are to be considered as any bias present in the training data will be replicated when determining pain. Following this, a wide range of participants should be considered as factors relating to age, gender, skin color, present comorbidities, etc., should be logged so a spectrum of variations can be accounted for before final pain analysis.

#### 6.2.3 Variability of anesthesia

Cortical Regions are a target for the hypnotic effects of many anesthetics, which are known to alter cerebral connectivity.^129^ While previous studies have shown that neurovascular coupling is intact across multiple anesthetics,^130^ in cases where anesthetics alter the brain metabolism and neurovascular coupling, for instance, in people with premorbid autonomic dysfunction, it would be possible to account for these differences by integrating simultaneous fNIRS-EEG systems. EEG systems measure the large-scale neuronal oscillatory activity from the cortical and subcortical regions. In its simplest form, we could use an fNIRS-EEG system, with electrodes placed on the forehead, to compute the depth of anesthesia, like a BIS. However, stand-alone fNIRS has also been able to differentiate the different phases of anesthesia reliably.^131^ Integrated fNIRS-EEG systems could help quantify both neuronal and neurovascular activity during surgery. Pain-related evoked potential (PREP), defined by negative and positive peaks of EEG activity following peripheral nociceptive stimulation, has been reported to be an objective assessment of nociceptive activity.^132^ ERPs are generally low in magnitude ∼0.1 to 1 microVolt, thus it is unclear whether these pain-related ERP features may be detected at a single-subject level.^133^ Furthermore, studies show that the N2 and P2 (the second negative and second positive peaks) components of PREP are associated with emotional valence and arousal to the nociceptive stimulation,^134^ both eliminated under anesthesia. Thus, further work is necessary to understand how ERP features may be integrated with fNIRS measures for real-time pain detection under anesthesia. Other practical limitations of EEG include greater susceptibility to motion, poor spatial resolution, and an extensive number of electrodes needed to achieve source localization, which can be challenging in surgery.

#### 6.2.4 Depth sensitivity

One of the recognized limitations of continuous wave fNIRS technique is the limited penetration depth, where only the hemodynamic changes in the superficial regions of the cortex (<1  cm) are quantifiable depending on the source-detector distance.^135^ Though our proposed approach does not depend on deep brain activity, the ability to capture and integrate deep brain activity into the algorithm could offer superior performance. Time-domain fNIRS (TD-fNIRS), another modality of fNIRS that employs time gating technology to discriminate the photons arriving at different penetrating depths, could possibly resolve this issue by providing up to 5 to 6 cm of depth penetration from the head surface and the ability to calculate absolute hemoglobin concentration and tissue oxygen saturation.^136^^,^^137^ The limiting factor, however, has been the practicality of this modality in a clinical setting; as the systems are bulky, computationally and operationally complex, with only one commercially available system.^137^ Despite these limitations, there are numerous studies involving in-house and commercial versions of the TD-fNIRS to quantify cerebral blood flow changes in the operating room during carotid artery repair, obstetrics surgery, and pregnancy complications.^138^ Given the advancements in laser/detector technology, TD-fNIRS could capture more cerebral hemodynamic, perfusion, and metabolism information than CW-fNIRS. Thus, future work should be directed at replicating and translating the current evidence captured using CW devices to a TD-based implementation.

#### 6.2.5 Issues pertaining to somatotopy and measures over the primary sensory cortex

One of the issues relating to measures of sensory changes is the relationship of the probes to the sensory homunculus that represents the map of major input from specific peripheral end organs to the higher-order cortical region (somatotopy). The placement of the probes covers the sensory cortex with the exception of the foot/toes, which dip into the medial part of the cortex. While major inputs correlate with the overall homunculus as shown by cortical mapping using electrophysiology,^72^^,^^139^ EEG^140^ and fMRI,^141^ the interactions are more complex and integrated.

The representations derived from somatic tissues (skin, muscles, tendons, and joints) are not specific or focal as noted by plasticity of these regions in development and functional use^142^^–^^144^ and following amputation or stroke.^145^ In support of this, widespread activation is noted that coexists with localized representation of a body part.^146^^–^^148^ Furthermore, recent data suggests that while primary activation is focused on different regions, there is also activation in non-dominant cortical regions including the secondary somatosensory cortex, which may also be bilateral.^149^^,^^150^ With visceral nociception does not have the same level of somatotopy activation in the paraylvian region of SI is present in experimental studies in humans (e.g., esophageal stimulation^151^ as well as activation in the same regions of the prefrontal cortex.^82^

While visceral sensation is represented in the insular cortex, there are major connections with the frontal lobes^152^ as there are from primary somatosensory regions, independent of other afferent inputs processing cognitive and affective information.^14^ As noted above, and from prior work, both experimental and clinical pain results in activation in the frontopolar cortex,^19^ a dominant region being measures in our fNIRS approach.

#### 6.2.6 Real-time applications of fNIRS

Offline analysis of NIRS is most common due to the slow nature of the hemodynamic response and the confounding noise sources that require significant processing of the data. However, online or real-time use of NIRS is not a new concept. Online analysis is particularly useful in brain–computer interfaces and NIRS is an excellent option for BCI due to its moderate spatial resolution, high temporal resolution and flexibility to be integrated with EEG. Several real-time adaptations of NIRS for BCI exist in the literature using classifiers such as multiple linear classifier,^153^ support vector machines,^154^ deep learning,^155^^,^^156^ etc. Real-time fMRI along with real-time fNIRS has been used as BCI neural feedback system to provide motor training in stroke survivors.^157^ Though computationally complex, real-time processing of NIRS is accessible to non-experts using Turbo-satori toolbox by NIRx Inc.^158^ More recent efforts have focused on improving the accuracy of real-time NIRS analysis. Ortega-Martinez and colleagues have demonstrated the use of Kalman filtering^159^ by means of a temporally embedded canonical correlation analysis to perform physiological noise correction of fNIRS data to improve online classification accuracy.^160^ Another group have proposed the use of moving average convergence divergence for noise reduction in NIRS data for online processing.^161^

#### 6.2.7 Other signal processing method considerations

In addition to the proposed FFT and low-frequency hemodynamic oscillation metrics, wavelet-based time-frequency analysis could be useful in comparing the brain and physiological response during ongoing nociceptive stimuli. Application wise, the process has degrees of flexibility to it, as it can often be computationally intensive compared to a simple cross-correlation or power spectrum analysis. However, it could provide important insights into how physiological response and brain response may potentially synchronize during nociceptive events and non-nociceptive events, like those performed in hyperscanning^162^^,^^163^ and SPA-fNIRS studies.^164^

#### 6.2.8 Will fNIRS measures improve short- and long-term outcomes?

The deployment of the system through wireless communication could theoretically mean that it can be performed for multiple patients simultaneously. An example of a multi-patient pain monitoring dashboard is shown in Fig. S7 in the Supplementary Material, where the user (i.e., clinician) may enter patient specific priors such as sex, baseline parameters, anesthetic induction, analgesic dose, etc. into the framework. Although, more work is needed to identify what confounds are needed to optimize model performance. Though we have defined a particular classifier approach, ideally one would have to evaluate multiple classifier performances, and validate and fine-tune the model parameters.

#### 6.2.9 Predictive value

**Fig. 7:**
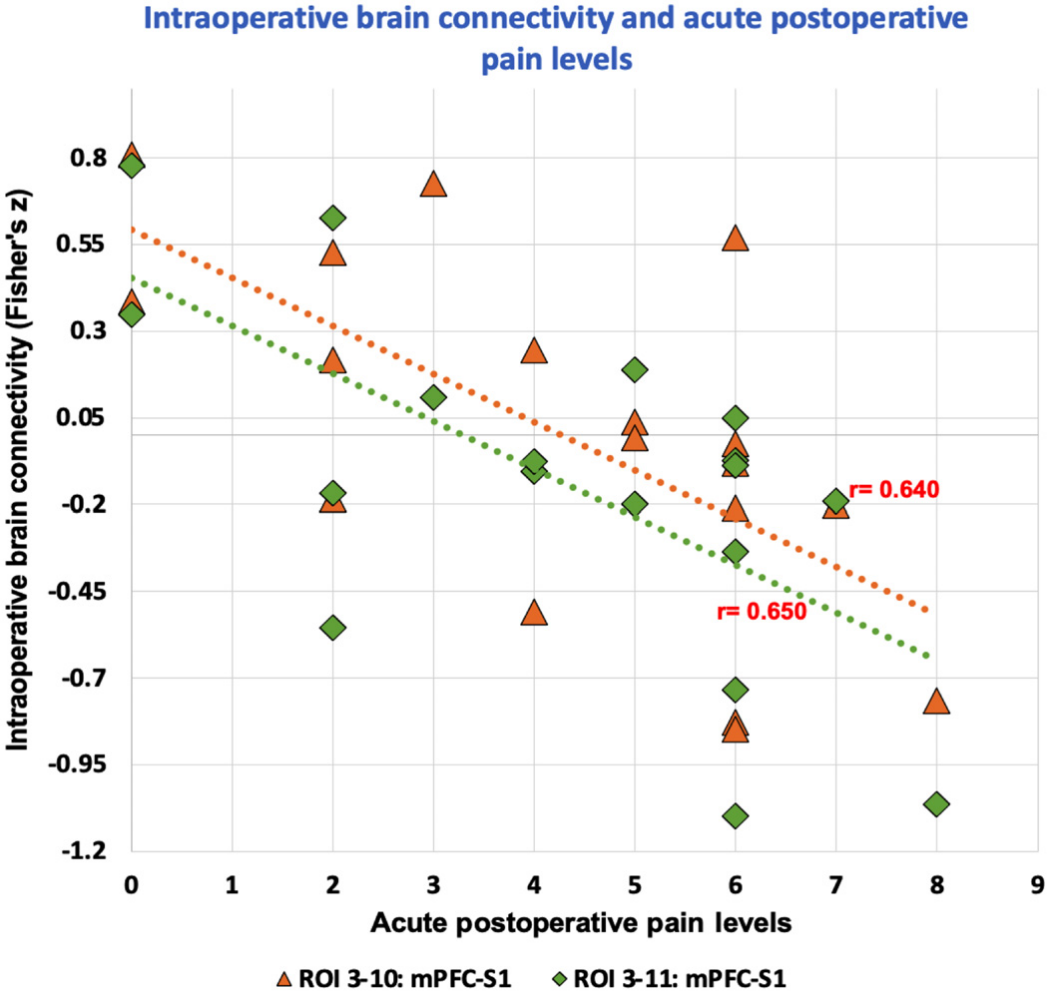
Prediction: Negative relationship between intraoperative cortical connectivity of mPFC and S1 regions during surgical procedures and pain levels in the post anesthesia unit. Higher negative connectivity between the two cortical regions, likely indicative of the nociceptive barrage, was associated with greater pain immediately after surgery (Data from Karunakaran et al.^104^).

The use of an objective measure has the possibilities in a number of areas related to effective treatment providing improved outcomes. These include (1) immediate response to pain/nociception or keeping pain/nociception low on an ongoing basis. As evidenced by a number of clinical parameters, either combined/multimodal anesthesia techniques^165^ or use of higher doses of intraoperative analgesia are associated with decreased levels of chronic pain as well as analgesic use;^166^ (2) prevention of central sensitization at the time of injury (an unusual opportunity or circumstance in the chronic pain domain) may contribute to the decrease of the acute to chronic pain transition.^167^^,^^168^ Such changes may have a neurobiological underpinning in preventing “neuronal ensembles” from becoming dysfunctional and contribute to enhanced processes in the acute to chronic pain transition (e.g., hyperaglgesia and anxiety);^169^ (3) premorbid conditions (e.g., catastrophizing) defined prior to surgery are known to predict poorer outcomes. Such conditions may enhance brain responsivity to trauma during the surgical procedure as well as pain levels immediately following surgery (Fig. 7). Recent findings from our group show that increased negative connectivity between mPFC and S1 in response to surgical procedures was negatively correlated with pain levels immediately after surgery.^104^ Intraoperative pain monitoring using NIRS may help predict both acute and chronic postsurgical pain outcomes.

#### 6.2.10 Drug overdose/excessive administration of analgesia

While we have focused on the benefits of utilization of fNIRS to provide a quantitative measure of pain/nociception, fNIRS has applications related to mitigating negative outcomes such as excessive drug administration. Drugs utilized in the operating room include opioids and inhalational anesthetics that may have deleterious effects on brain function when given in overdose. Such events may contribute to prolonged emergence and other complications such as emergence delirium.^170^ EEG has been used to evaluate anesthesia dosing^171^ including anesthetic depth^172^ although some caution the sensitivity of EEG measures for depth monitoring.^173^ “Since no ‘gold-standard’ method is available to continuously, reliably, and effectively monitor the effects of sedatives and anesthetics, such a method is greatly needed.”^174^ fNIRS has been used to evaluate anesthetic depth resulting from either ongoing administration of drugs or bolus effects of drugs on brain function^174^^,^^175^ and data support the use of fNIRS in accurately evaluating classification of the anesthetized state.^175^

#### 6.2.11 Surgical awareness

Intraoperative or surgical awareness, which is estimated to affect between 1:1000 and 1:20,000 patients “is characterized by the coincidence of both intraoperative consciousness and explicit, episodic postoperative recall of events during a planned anesthetic.”^176^ As a result, patients may suffer pain as well as psychological trauma from the event. It may be present when with neuromuscular blockade or insufficient anesthesia. The issue of “subconscious pain” has not been easily evaluated when analgesics are not provided. While fNIRS has not been used for anesthetic depth, the fNIRS measures could easily have added measures/algorithms to signal potential awareness through a combination of signals for pain and auditory measures as described in sections above. In addition, fNIRS can measure motor activity,^177^^,^^178^ even imagined motor activity,^179^ or patients in a locked in state (i.e., awake but cannot communicate^180^). In the “paralyzed/awake state,” increased activity in the motor cortex would be expected due to “escape” or attempt to motion or speak.

## 7 Conclusions

### 7.1 Need for a Biomarker

Currently, there is no biomarker for acute or ongoing pain that can be easily used in the clinic; and the evaluation of efficacy of therapeutic interventions (drug or behavioral or interventional) is an issue. If the biomarker is proven in its efficacy, it will certainly have a good chance of adoption in the clinic, clinical trials, and potentially in new approaches for evaluating treatment efficacy in the clinic. While anesthesia provides a state of unconsciousness, there are no objective measures of evoked or ongoing pain (i.e., analgesia) while under anesthesia. The lack of analgesic control may induce a response in the brain called central sensitization that is the harbinger of two main deleterious outcomes: (1) increased pain and use of opioids in the postoperative period and (2) the initiation of a chronic neuropathic pain process (that may also be driven by the nerve damage from the surgical incisions – peripheral sensitization).

As noted in the introduction the notion that during anesthesia ongoing nociceptive activation can impact brain systems is perhaps not intuitive. As noted above, accumulating evidence suggests that peripheral activation of nociceptors may activate brain systems under general anesthesia including but not limited to (1) brain responses occur to evoked pain under anesthesia in animal models; (2) while under general anesthesia there is a preservation of lower order sensory networks, including thalamo-cortical connectivity, even at anesthetic concentrations that suppress responsiveness; (3) preoperative pain or preoperative pain sensitivity predicts increased postoperative pain and analgesic requirements and long term persistent pain, suggesting a process akin to central sensitization during surgery. Thus, the concept of evoked and ongoing pain (pain load) in patients undergoing surgery under general anesthesia is a concept that needs to be challenged because of the consequences of increased postoperative pain, increased analgesic use and potential pain chronification.

### 7.2 Older Technologies Do Not Measure Nociception/Pain

The BIS monitor is the only system in regular use that provides some assessment of the depth of anesthesia.^181^ Other systems deploying autonomic measures are also being developed for use of measure of pain in the OR. Whether BIS constitutes an accurate measure of the depth of anesthesia or not, it certainly does not aid the anesthesiologist in the evaluation of pain. fNIRS can potentially provide an objective, brain function derived, quantified assessment of intra-operative pain. The successful outcome of this project would provide a new patient monitoring device that may be as transformative as the pulse-oximeter. Thus, the need is obvious, the imperative to evaluate pain in the OR might provide significant benefits to patients through a “nociceptive free” unconscious experience. fNIRS can also be used to determine the depth of anesthesia.^174^

### 7.3 Societal Benefits

The technological development will provide an objective readout of pain state and therefore has the potential to standardize the evaluation of pain and the effect of analgesics in the clinic and in clinical trials, and across populations (i.e., adults and children). Such an advance would end the empirical, subjective assessment of pain and pain killers, would improve the efficiency and reliability of clinical trials and would therefore decrease the number of subjects exposed to experimental analgesic drugs. The clinical implementation/use of fNIRS for pain measures may also result in a number of other potential applications including the assessment of pain in patients who cannot effectively communicate (such as infants and the physically or neurologically impaired) and the creation of an automated, self-regulating pain control system for post-operative patients or mass evacuation of injured persons (e.g., in the military transport of wounded warriors). In order to be successful, the measure should have high specificity and sensitivity; easy to implement – including testing procedures and evaluation processes (i.e., analysis, interpretation); and the cost-benefit should be good and adoption by the specific community (i.e., surgeons or anesthesiologists) is essential.

## Supplementary Material



## Data Availability

Data sharing is not applicable to this article as no new data were created or analyzed.
