# Supplementary material for: Machines, mathematics, and modules: the potential to provide real-time metrics for pain under anesthesia
Source: Neurophotonics. 2024 Feb 22;11(1):010701. doi: 10.1117/1.NPh.11.1.010701 (PMC10883389; doi:10.1117/1.NPh.11.1.010701)
Supplement: Supplementary file 1 [file NPh_011_010701_SD001.pdf]

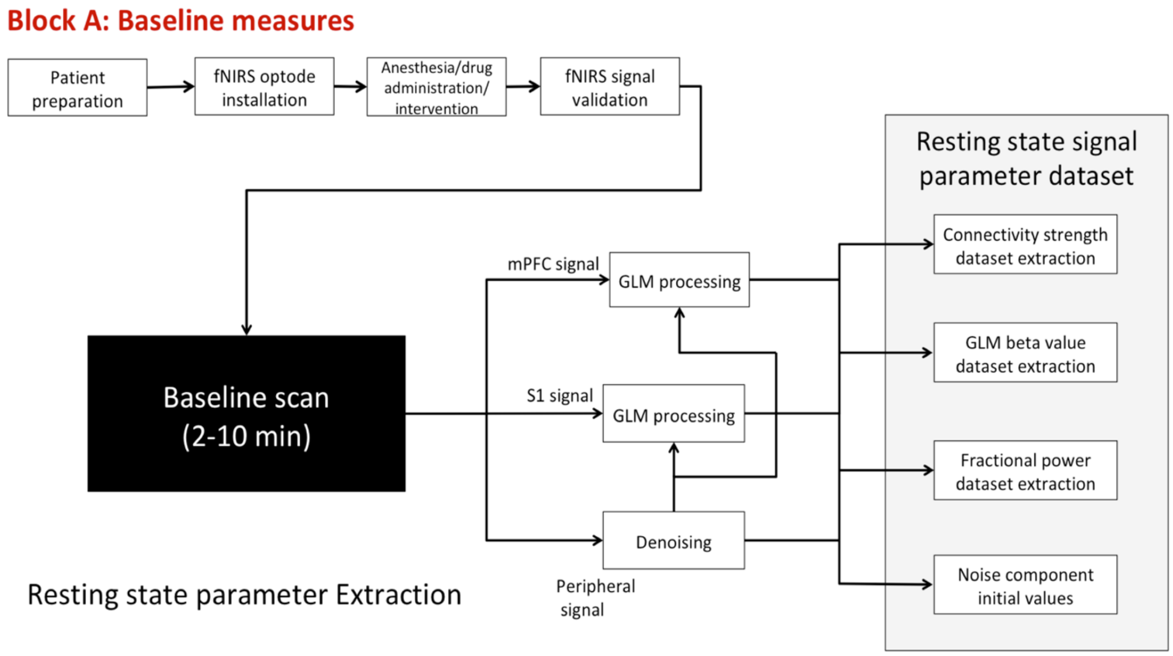

Figure S1. Block A: Procedures of conducting baseline data acquisition and analysis to set up a dataset of resting state (baseline) signal parameters. These include the installation of fNIRS cap and optodes, signal validation and adjustment, a baseline scan of 2-10 minutes, and a standard GLM analysis of the obtained baseline data to extract a dataset of signal parameters (functional connectivity strength, GLM beta values, mPFC signal fractional power and noise level) in a resting state condition for a specific patient.

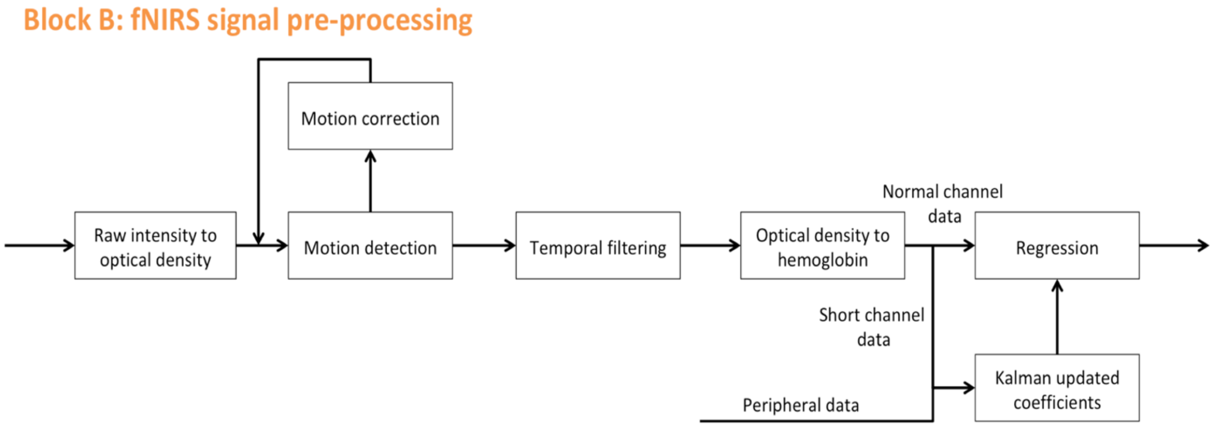

Figure S2: Block B: Pre-processing of recorded fNIRS buffered data. The raw fNIRS optical data are first converted into optical density values. Motion detection and correction are applied to correct sudden drifts and spikes in the time course coming from patient movement or optode displacement. The cutoff frequencies of the band-pass temporal filters are 0.01Hz and 0.5Hz. After calculating hemoglobin concentration changes, the short channel regression method is applied to remove the systemic physiological noises from superficial head layers such as the scalp or the skull.

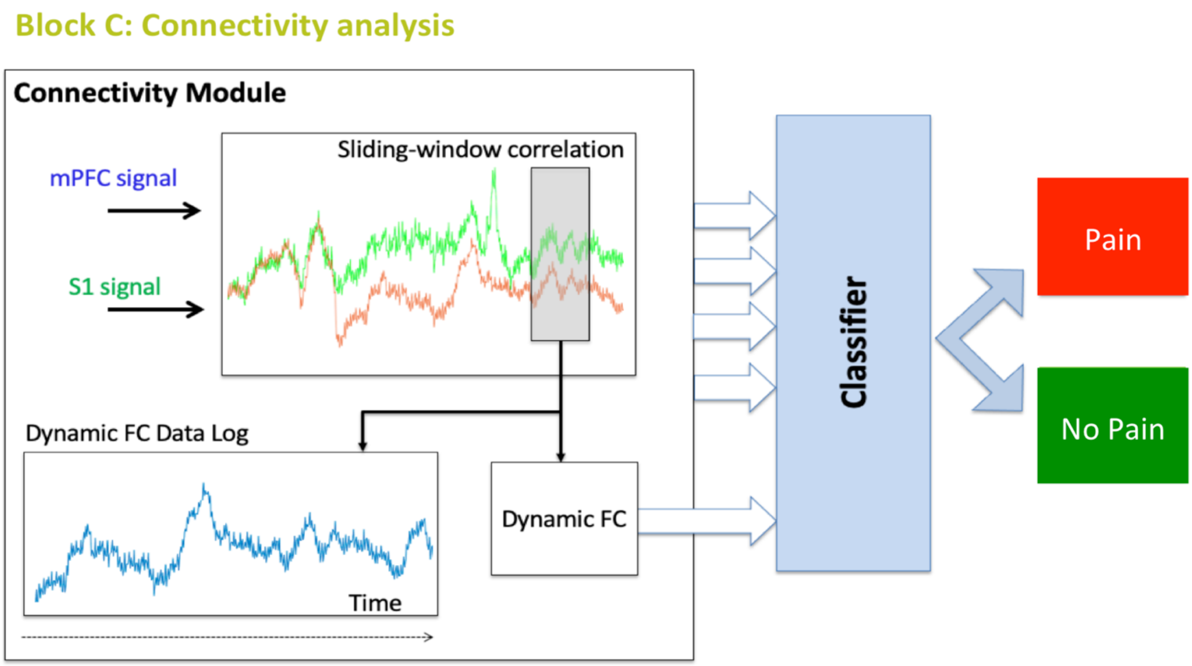

Figure S3: Block C: Dynamic Functional connectivity module to extract connectivity-based features as input for Classifier. A sliding window correlation can be used to evaluate the pairwise correlation between mPFC and S1 signals in short windows over time. The output correlation measure represents the dynamic connectivity measure.

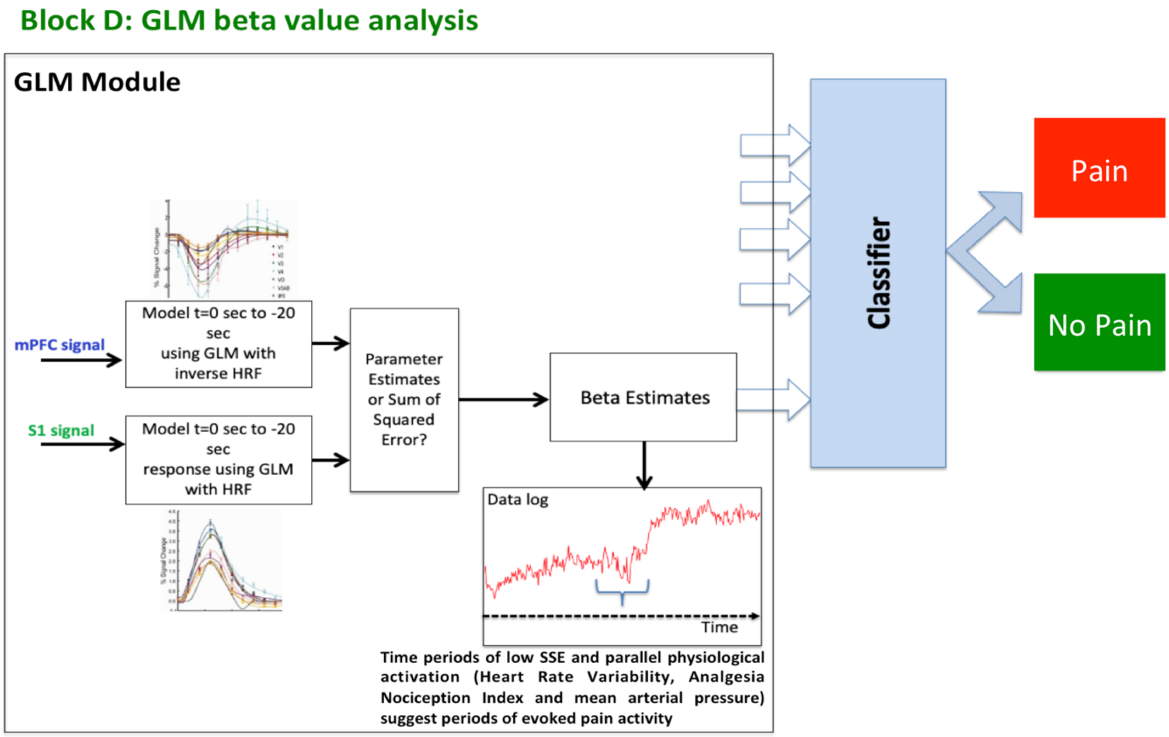

Figure S4: Block D: GLM beta value analysis. GLM module to extract model-based features as input for Classifier. A canonical HRF can be used to model the variance of mPFC and S1 signals in 20 second windows over time. Comparison of the estimates from mPFC and S1 together can be used to define a nociception index.

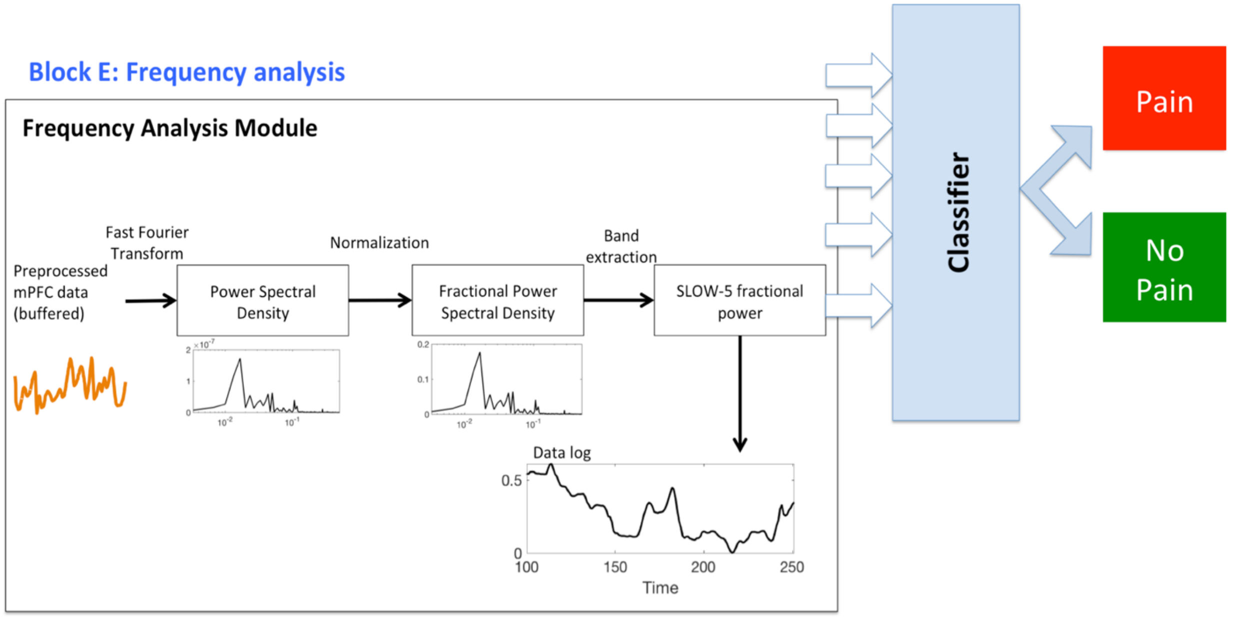

Figure S5: Block E: Frequency analysis of power shifts in fNIRS signal low frequency oscillations. The buffered mPFC data are first transformed into power spectral density measurements with a fast Fourier Transform. After normalization, the fractional power of the slow-5 band (0.01-0.027Hz) is extracted and inputted to the classifier for ongoing pain classification (Peng et al., 2021).

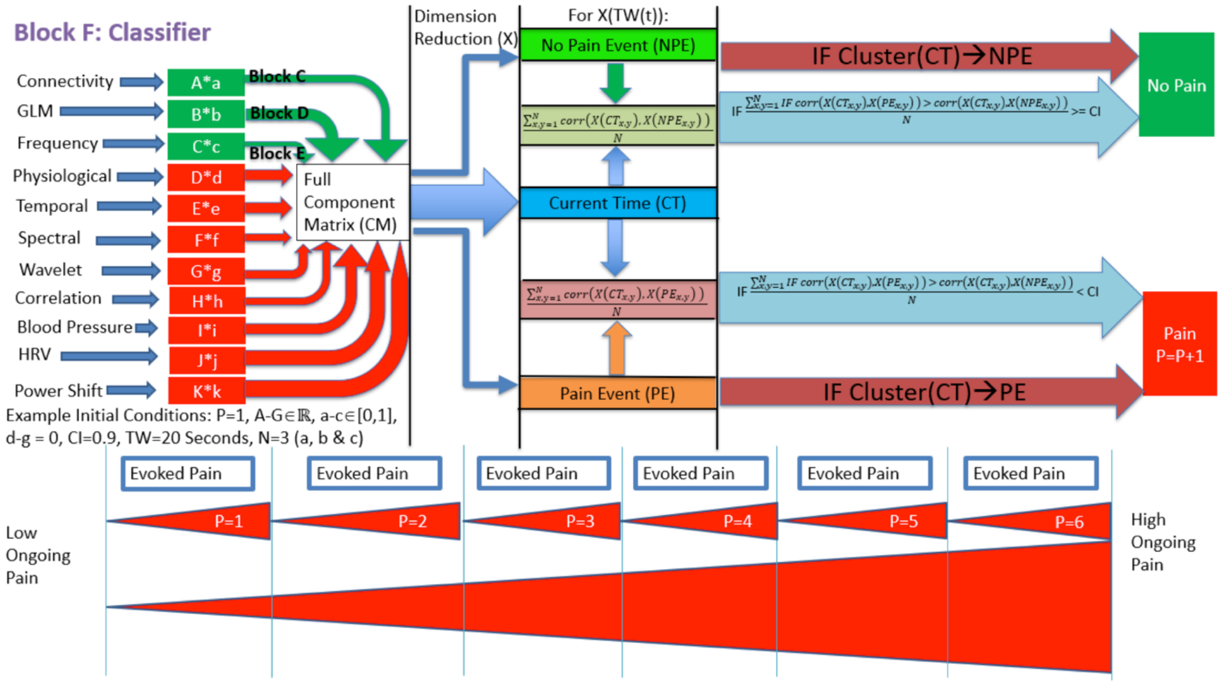

Figure S6: Block F: Pain/No Pain classifier design. A surgeon wants to use Connectivity Analysis, General Linear Model Beta Analysis and Frequency as measures to calculate when a patient is undergoing pain during surgery (see blocks C, D and E). The resulting matrices A, B and C are multiplied by a, b c, and c respectively that correctly weigh the relevance of each variable to the pain reading. These vectors then form a component matrix CM that undergoes dimension reduction such as Principal Component Analysis and the resulting components form the matrix X. X is calculated at the baseline where no procedures are carried out (NPE), the current point in time (CT) and for at least one pain procedure externally recorded by the surgeon/anesthesiologist/clinical staff (PE) for TW seconds (in this example, TW=20). The set of pain procedures recorded PE and the values recorded at the baseline NPE form two clusters which can classify the current point of time t. Alternatively, if 90% or more of the correlations are greater between the variables in CT and PE than between CT and NPE, this can be registered as a pain event and the pain counter can be updated. If the clustering or correlation conditions are not met, then no further action is taken regardless of method.

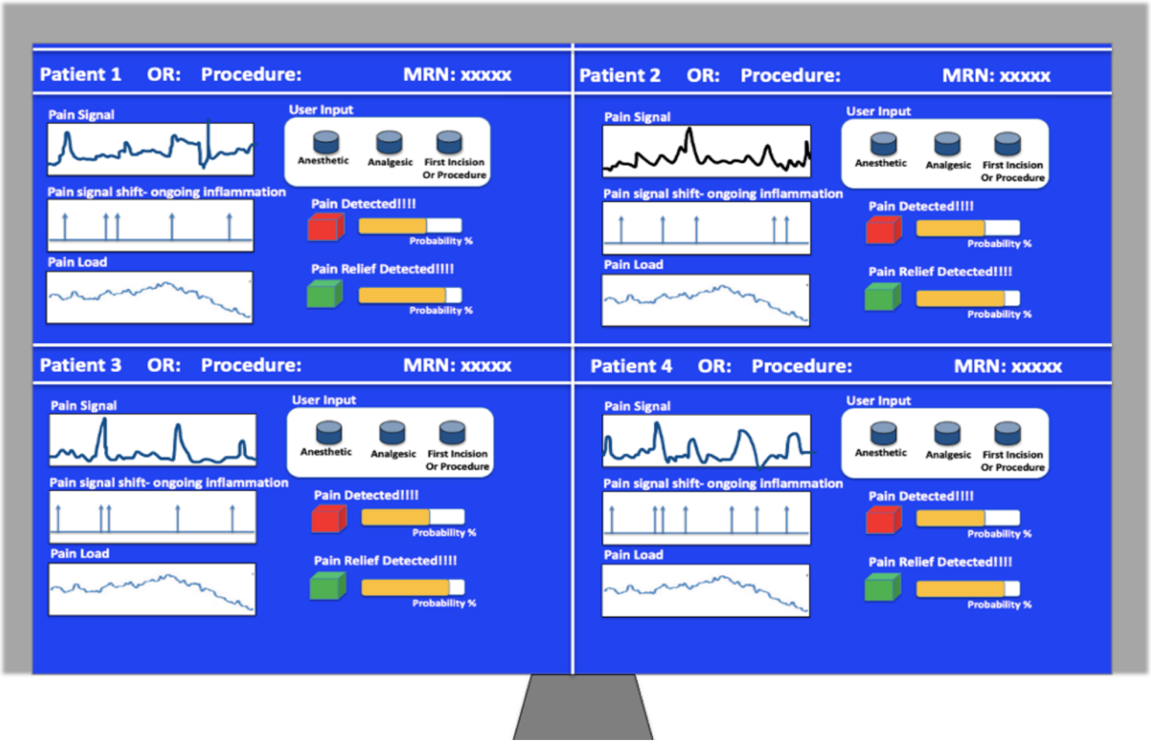

Figure S7: An example of a real-time multi-patient monitoring dashboard that indicates "Pain Detected" or "No Pain Detected".
